# Impaired Metabolomics of Sulfur-Containing Substances in Rats Acutely Treated with Carbon Tetrachloride

**DOI:** 10.5487/TR.2008.24.4.281

**Published:** 2008-12-01

**Authors:** Sun Ju Kim, Do Young Kwon, Kwon Hee Choi, Dal Woong Choi, Young Chul Kim

**Affiliations:** 16 College of Pharmacy, Seoul National University, San 56-1 Shinrim-Dong, Kwanak-Ku, Seoul, 151-742 Korea; 26 College of Health Science, Korea University, Seoul, 136-703 Korea; 36 Research Institute of Pharmaceutical Sciences, Seoul National University, Seoul, 151-742 Korea

**Keywords:** Carbon tetrachloride, Transsulfuration, S-adenosylmethionine, Glutathione, Taurine

## Abstract

Impairment of hepatic metabolism of sulfur-containing amino acids has been known to be linked with induction of liver injury. We determined the early changes in the transsulfuration reactions in liver of rats challenged with a toxic dose of CCl_4_ (2 mmol/kg, ip). Both hepatic methionine concentration and methionine adenosyltransferase activity were increased, but S-adenosylmethionine level did not change. Hepatic cysteine was increased significantly from 4 h after CCl_4_ treatment. Glutathione (GSH) concentration in liver was elevated in 4~8 h and then returned to normal in accordance with the changes in glutamate cysteine ligase activity. Cysteine dioxygenase activity and hypotaurine concentration were also elevated from 4 h after the treatment. However, plasma GSH concentration was increased progressively, reaching a level at least several fold greater than normal in 24 h. γ-Glutamyltransferase activity in kidney or liver was not altered by CCl_4_, suggesting that the increase in plasma GSH could not be attributed to a failure of GSH cycling. The results indicate that acute liver injury induced by CCl_4_ is accompanied with extensive alterations in the metabolomics of sulfur-containing amino acids and related substances. The major metabolites and products of the transsul-furation pathway, including methionine, cysteine, hypotaurine, and GSH, are all increased in liver and plasma. The physiological significance of the change in the metabolomics of sulfur-containing substances and its role in the induction of liver injury need to be explored in future studies.

## INTRODUCTION

Carbon tetrachloride ($CCl_4$) is a model hepatotoxicant. The induction of $CCl_4$ toxicity is attributed to generation of a free radical, $\cdot CCl_3$, by enzyme reactions mediated mostly by cytochrome P450 2E1. The free radical binds covalently to lipids and proteins, resulting in structural damage of membranes and a variety of enzymes. The reactive species also initiates lipid peroxidation by attacking enoic fatty acids leading to generation of organic free radicals, which may in turn react with $O_2$ to form peroxides and other cytotoxic metabolites (Recknagel *et al.*, 1989).

In mammals the liver plays a central role in the sulfur-amino acid metabolism (Mudd and Poole, 1975). Sulfur-amino acid metabolism occurs primarily via the transsulfuration pathway, which results in transfer of sulfur from methionine to serine to form cysteine. The first step in methionine metabolism is formation of S-adenosylmethionine (SAM) that is catalyzed by methionine adenosyltransferase (MAT). SAM serves as a methyl donor for various biological methylation reactions, and the co-product of transmethylation, S-adenosylhomocysteine (SAH), is hydrolyzed to yield homocysteine which is either remethylated to methionine or condensed with serine into cystathionine. Condensation of homocysteine and serine to cystathionine and subsequent release of cysteine from cystathionine are mediated by cystathionine β-synthase (CβS) and cystathionine γ-lyase (CγL), respectively. Cysteine is metabolized in liver to yield either taurine, inorganic sulfate, or glutathione (GSH). Cysteine dioxygenase (CDO) catalyzes oxidation of this amino acid to cysteinesulfinate that is converted to taurine via hypotaurine. Synthesis of GSH from cysteine is mediated by glutamate cysteine ligase (GCL) and GSH synthetase, consecutively.

The intermediates or final products in the transsulfuration reactions, including SAM and GSH, have essential roles in the maintenance of the normal biochemistry and physiology of mammalian livers. In this study we examined the changes in hepatic metabolism of sulfur-amino acids in acute liver injury induced by $CCl_4$ treatment. Despite the well-known hepatotoxic potential of this organic solvent, its effects on the hepatic transsulfuration reactions have hardly been studied. Considering the devastating effects of $CCl_4$ on the liver, it is suspected that significant alterations in the metabolomics of sulfur-containing substances would take place, which may encompass important toxicological implications in liver. Since the hepatotoxicity of $CCl_4$ is primarily associated with lipid peroxidation, examination of its effects on the transsulfuration pathway that supplies GSH, a major antioxidant in liver, as the final product was of particular interest.

## MATERIALS AND METHODS

***Animals and treatments.*** Male Sprague-Dawley rats were purchased from Dae-Han Laboratory Animal (Seoul, Korea). The use of animals was in compliance with the guidelines established by the Animal Care Committee of this institute. Rats were housed in temperature (22 ± 2°C) and humidity (55 ± 5%) controlled rooms with a 12 h light/dark cycle. Laboratory rat chow and tap water were allowed *ad libitum*. Rats, weighing 250~300 g, were fasted from 24 h before $CCl_4$ treatment. $CCl_4$ diluted in corn oil was administered intraperitoneally to rats at a dose of 2 mmol/kg. Control rats received an equivalent volume of corn oil.

***Determination of liver and kidney toxicity.*** Blood was sampled from abdominal aorta in rats under light ether anesthesia. Activities of aspartate aminotransferase (AST) and alanine aminotransferase (ALT) in serum were determined by the method of Reitman and Frankel (1957). Serum creatinine (sCr) and blood urea nitrogen (BUN) were measured using commercially available kits (Youngdong Pharmaceutical, Seoul, Korea).

Malondialdehyde (MDA) in liver was quantified using the method of Volpi and Tarugi (1998). A HPLC system equipped with a fluorescence detector and a 5 μm Symmetry C18 reversed phase column (4.6 × 150 mm; Eka, Bohus, Sweden) was used. The mobile phase was composed of 35% methanol and 65% 50 mM sodium phosphate buffer, pH 7.0.

***Determination of sulfur-containing metabolites.*** Liver was homogenized in a fourfold volume of cold 1 M perchloric acid. After centrifugation, GSH concentration in the supernatant was determined using an enzymatic recycling method (Griffith, 1980). Cysteine level was estimated by the acid-ninhydrin method (Gaitonde, 1967). The method of She *et al.* (1994) was employed to determine SAM and SAH concentrations. The supernatant was directly applied to a HPLC system with a UV detector and a TSK-GEL ODS-80TM column (4.6 × 250 mm) (Tosoh, Tokyo, Japan).

Liver was homogenized in a fivefold volume of cold methanol for analysis of methionine, hypotaurine and taurine. Serum was diluted with methanol. Methionine, hypotaurine and taurine were derivatized with *O*-phthalaldehyde/2-mercaptoethanol and quantified using HPLC with a fluorescence detector and a 3.5 μm Kromasil C18 column (4.6 × 100 mm) (Eka, Bohus, Sweden). Methionine was measured using the method of Rajendra (1987). The method of Ide (1997) was used to separate hypotaurine and taurine.

***Enzyme assays.*** Liver homogenate in a threefold volume of ice-cold buffer consisting of 0.154 M KCl/ 50 mM Tris-HCl and 1 mM EDTA (pH 7.4) was centrifuged at 10,000 g for 20 min. The supernatant was further centrifuged at 104,000 g for 60 min. The 104,000 g supernatant fraction (cytosol) was used to determine the enzyme activities.

The activity of MAT was estimated by quantifying SAM and SAH production as described above. The reaction mixture consisted of 80 mM Tris-HCl/50 mM KCl (pH 7.4), 5 mM ATP, 40 mM $MgCl_2$, 0.1 mM methionine, and enzyme solution containing 1 mg protein in a final volume of 1 ml. The incubation was carried at 37°C for 30 min. CβS activity was determined by quantifying generation of cystathionine (Kashiwamata and Greenberg, 1970). The method of Matsuo and Greenberg (1957) was used to determine CγL activity. GCL activity was determined using the method of Sekura and Meister (1977). Formation of γ-glutamylcysteine was quantified after *O*-phthalaldehyde derivatization according to a HPLC separation/fluorometric detection method (Yan and Huxtable, 1995). The method of Bagley *et al.*

(1995) was used to measure CDO activity. The amount of cysteinesulfinate formed was quantified by a HPLC system equipped with a fluorescence detector and a 3.5 μm Kromasil C18 column (4.6 × 100 mm) after *O*-phthalaldehyde/2-mercaptoethanol derivatization.

The activity of γ-glutamyltransferase (GGT) was measured using whole tissue homogenates. Renal GGT activity was assayed kinetically at 37°C by the method of Orlowski and Meister (1963). γ-Glutamyl-*p*-nitroanilide was used as a substrate. A non-kinetic method was used to determine hepatic GGT activity (Hinchman and Ballatori, 1999). Tissue homogenates and the substrate were added to a reaction mixture containing Tris-glycine buffer (pH 8.0) for incubation at 37°C for 30 min.

***Data analysis.*** All results expressed as the mean ± S.E.M. were analyzed by a two-tailed Student's *t*-test. The acceptable level of significance was established at $P < 0.05$ except when otherwise indicated.

## RESULTS

***$CCl_4$-induced liver and kidney toxicity.*** Serum AST and ALT activities were elevated significantly from 4 h following $CCl_4$ treatment (Table 1). The enzyme activities in serum were increased steadily for 24 h after the treatment. Hepatic MDA levels were increased rapidly after $CCl_4$ treatment, but returned to normal at t = 8 h. The increase in lipid peroxidation preceded the elevation of serum AST and ALT activities, suggesting a causal role of lipid peroxidation in $CCl_4$-induced hepatotoxicity. The dose of $CCl_4$ used in this study also induced renal injury. Both BUN and sCr were elevated at t = 8 h, which remained at a level higher than control till 24 h after $CCl_4$ treatment.

***Concentration of sulfur-containing metabolites in liver.*** Changes in hepatic concentrations of sulfur-containing substances are summarized in Table 2. Methionine was not changed immediately after $CCl_4$ treatment,

**Table 1.** Changes in parameters of liver and kidney toxicity in rats treated with $CCl_4$

| Time | Group | ALT | AST | MDA | sCr | BUN |
|---|---|---|---|---|---|---|
| | | units/ml serum | | nmol/g liver | mg/dl serum | |
| 0 h | Control | 34.5 ± 2.0 | 79.9 ± 1.0 | 3.34 ± 0.36 | 0.30 ± 0.02 | 19.1 ± 1.1 |
| 2 h | Vehicle | 34.4 ± 2.2 | 95.1 ± 7.5 | 3.71 ± 0.26 | N/D | N/D |
| | $CCl_4$ | 36.4 ± 2.4 | 112.3 ± 7.5 | 5.82 ± 0.25** | N/D | N/D |
| 4 h | Vehicle | 33.1 ± 3.1 | 89.2 ± 7.7 | 4.45 ± 0.17 | N/D | N/D |
| | $CCl_4$ | 273.2 ± 39.4** | 384.5 ± 48.9** | 5.91 ± 0.35** | N/D | N/D |
| 8 h | Vehicle | 32.2 ± 3.0 | 88.5 ± 6.5 | 4.84 ± 0.23 | 0.31 ± 0.02 | 16.9 ± 0.9 |
| | $CCl_4$ | 629.8 ± 135.8** | 596.2 ± 96.5** | 4.93 ± 0.21 | 0.46 ± 0.05* | 19.9 ± 0.7* |
| 24 h | Vehicle | 32.8 ± 4.4 | 79.8 ± 5.5 | 5.31 ± 0.36 | 0.30 ± 0.03 | 16.1 ± 0.6 |
| | $CCl_4$ | 1564.9 ± 177.8** | 1980.6 ± 397.2** | 4.92 ± 0.19 | 0.41 ± 0.03* | 22.2 ± 2.1* |

Rats were treated intraperitoneally with $CCl_4$ (2 mmol/kg). Each value represents mean ± S.E.M. for 4 or 5 rats. *,**Significantly different from the rats treated with vehicle only at $P < 0.05$, 0.01, respectively. N/D; not determined.

**Table 2.** Changes in sulfur-containing substances in liver of rats treated with $CCl_4$

| Time | Group | Methionine | SAM | SAH | Cysteine | GSH | Hypotaurine | Taurine |
|---|---|---|---|---|---|---|---|---|
| | | nmol/g liver | | | | μmol/g liver | | |
| 0 h | Control | 64.0 ± 3.4 | 52.1 ± 2.6 | 23.9 ± 2.3 | 109 ± 4.4 | 4.42 ± 0.09 | 0.22 ± 0.02 | 1.95 ± 0.28 |
| 2 h | Vehicle | 64.8 ± 5.5 | 53.1 ± 2.5 | 23.5 ± 1.6 | 100 ± 9.6 | 4.42 ± 0.29 | 0.20 ± 0.03 | 5.20 ± 1.15 |
| | $CCl_4$ | 73.8 ± 4.3 | 52.2 ± 3.3 | 21.8 ± 0.4 | 87 ± 2.9 | 4.16 ± 0.09 | 0.31 ± 0.07 | 4.70 ± 0.84 |
| 4 h | Vehicle | 66.7 ± 2.2 | 48.6 ± 3.3 | 22.4 ± 1.1 | 85 ± 1.5 | 3.44 ± 0.11 | 0.16 ± 0.03 | 4.29 ± 0.55 |
| | $CCl_4$ | 77.3 ± 4.5 | 55.2 ± 1.1 | 19.2 ± 0.8 | 109 ± 4.8** | 4.79 ± 0.19** | 0.32 ± 0.03** | 2.61 ± 0.41* |
| 8 h | Vehicle | 72.2 ± 6.4 | 53.1 ± 2.2 | 24.3 ± 1.5 | 87 ± 3.2 | 3.43 ± 0.13 | 0.14 ± 0.02 | 5.31 ± 0.87 |
| | $CCl_4$ | 74.6 ± 2.2 | 54.5 ± 1.4 | 17.2 ± 0.9** | 108 ± 4.3** | 6.22 ± 0.26** | 0.19 ± 0.03 | 1.92 ± 0.32** |
| 24 h | Vehicle | 54.5 ± 5.1 | 53.3 ± 2.1 | 20.9 ± 0.9 | 86 ± 6.7 | 3.95 ± 0.20 | 0.09 ± 0.01 | 2.62 ± 0.61 |
| | $CCl_4$ | 92.1 ± 3.5** | 57.4 ± 1.5 | 17.8 ± 1.1 | 156 ± 9.6** | 4.52 ± 0.26 | 0.33 ± 0.10* | 2.13 ± 0.08 |

Each value represents mean ± S.E.M. for 4 or 5 rats. *,**Significantly different from the rats treated with vehicle only at $P < 0.05$, 0.01, respectively.

**Table 3.** Changes in sulfur-containing substances in plasma of rats treated with $CCl_4$

| Time | Group | Methionine | Cysteine | GSH | Taurine |
|---|---|---|---|---|---|
| | | nmol/ml plasma | | | |
| 0 h | Control | 51.9 ± 1.9 | 12.5 ± 0.4 | 3.54 ± 0.27 | 167 ± 14 |
| 2 h | Vehicle | 54.0 ± 1.5 | 12.6 ± 1.1 | 3.43 ± 0.19 | 144 ± 20 |
| | $CCl_4$ | 52.9 ± 2.4 | 10.1 ± 1.0 | 3.27 ± 0.18 | 125 ± 11 |
| 4 h | Vehicle | 52.1 ± 2.4 | 11.3 ± 0.6 | 3.19 ± 0.29 | 157 ± 12 |
| | $CCl_4$ | 57.5 ± 3.8 | 8.9 ± 0.4* | 5.23 ± 0.38** | 238 ± 11** |
| 8 h | Vehicle | 41.6 ± 2.0 | 10.7 ± 1.1 | 2.75 ± 0.14 | 148 ± 15 |
| | $CCl_4$ | 48.5 ± 1.5* | 11.9 ± 0.7 | 8.52 ± 1.49** | 179 ± 4 |
| 24 h | Vehicle | 48.2 ± 1.1 | 8.1 ± 0.7 | 3.20 ± 0.37 | 177 ± 27 |
| | $CCl_4$ | 73.0 ± 1.5** | 13.5 ± 1.2** | 30.95 ± 3.55** | 299 ± 42* |

Each value represents mean ± S.E.M. for 4 or 5 rats. *,** Significantly different from the rats treated with vehicle only at $P < 0.05$, 0.01, respectively.

**Table 4.** Changes in enzyme activities involved in the sulfur-containing amino acid metabolism in liver of rats treated with $CCl_4$

| Time | Group | MAT | CβS | CγL | GCL | CDO | GGT | |
|---|---|---|---|---|---|---|---|---|
| | | product formed (nmol)/min/mg protein | | | | | nmol/min/g liver | μmol/min/g kidney |
| 0 h | Control | 0.18 ± 0.01 | 6.94 ± 0.27 | 68.43 ± 3.45 | 5.57 ± 0.22 | 0.029 ± 0.001 | 163 ± 22 | 259 ± 5 |
| 2 h | Vehicle | 0.20 ± 0.02 | 8.47 ± 0.51 | 73.03 ± 5.87 | 6.53 ± 0.65 | 0.027 ± 0.002 | N/D | N/D |
| | $CCl_4$ | 0.20 ± 0.02 | 6.21 ± 0.52* | 66.48 ± 1.19 | 7.22 ± 0.98 | 0.026 ± 0.001 | N/D | N/D |
| 4 h | Vehicle | 0.32 ± 0.03 | 6.38 ± 0.48 | 70.11 ± 2.78 | 6.93 ± 0.57 | 0.030 ± 0.003 | N/D | N/D |
| | $CCl_4$ | 0.56 ± 0.02** | 6.39 ± 0.72 | 69.41 ± 2.97 | 8.70 ± 0.61 | 0.057 ± 0.005** | N/D | N/D |
| 8 h | Vehicle | 0.50 ± 0.05 | 6.30 ± 0.30 | 60.57 ± 2.13 | 6.38 ± 0.66 | 0.028 ± 0.002 | 171 ± 16 | 273 ± 6 |
| | $CCl_4$ | 1.11 ± 0.21* | 5.79 ± 0.56 | 57.70 ± 2.23 | 10.45 ± 0.47** | 0.040 ± 0.002** | 166 ± 31 | 263 ± 4 |
| 24 h | Vehicle | 0.19 ± 0.01 | 5.29 ± 0.22 | 69.84 ± 1.18 | 7.00 ± 0.21 | 0.023 ± 0.001 | 117 ± 15 | 256 ± 7 |
| | $CCl_4$ | 0.27 ± 0.03* | 5.41 ± 0.24 | 44.06 ± 2.74** | 7.09 ± 0.66 | 0.022 ± 0.001 | 114 ± 10 | 241 ± 7 |

Each value represents mean ± S.E.M. for 4 or 5 rats. *,** Significantly different from the rats treated with vehicle only at $P < 0.05$, 0.01, respectively. N/D; not determined.

but increased significantly at t = 24 h. SAM and SAH levels appeared to be unaffected by the dose of $CCl_4$ administered. Cysteine was elevated gradually from 4 h after the treatment. Major metabolic products of cysteine catabolism, GSH and hypotaurine, were also elevated in liver from t = 4 h. On the contrary, hepatic taurine was decreased significantly from t = 4 to 8 h.

Plasma methionine and cysteine levels were changed in a manner similar to that shown in liver (Table 3). However, GSH levels in plasma, elevated from 4 h after the treatment, increased progressively, reaching approximately a tenfold of control in 24 h. Taurine was elevated from 4 h following $CCl_4$ treatment, although the magnitude of increase was much less than that of GSH.

***Enzyme activities involved in the transsulfuration reactions.*** Hepatic MAT activity was increased rapidly by $CCl_4$ treatment, which remained at a level significantly higher than control for 24 h (Table 4). MAT activity in control rats was changed significantly in 24 h, suggesting a circadian variation of this enzyme. CβS and CγL activities appeared to be slightly decreased. The activities of GCL and CDO, which have key roles in the conversion of cysteine to GSH and hypotaurine, were induced transiently, but significantly, and returned to normal in 24 h. GGT activity was greater in kidney than in liver by more than a thousand-fold. Neither the liver GGT activity nor the renal GGT activity was altered by a hepatotoxic dose of $CCl_4$.

## DISCUSSION

It has long been realized that chronic liver injury is often associated with impairment of sulfur-amino acid metabolism (Kinsell *et al.*, 1947; Horowitz *et al.*, 1981). A link between ethanol-induced elevation of homocysteine and endoplasmic reticulum (ER) stress has also been demonstrated (Ji *et al.*, 2004). Further studies have shown that an elevation of homocysteine in liver may trigger activation of hepatic stellate cells through oxidative stress, which ultimately results in hepatic fibrosis (Adinolfi *et al*., 2005). Conversely, metabolic intermediates or products in the transsulfuration reactions, such as SAM, S-methylthioadenosine and taurine, were shown to be effective against induction of hepatic fibrosis in experimental animals (Simile *et al*., 2001; Nieto and Cederbaum, 2005; Miyazaki *et al*., 2005). This suggests a possibility that disturbance of the metabolomics of sulfur-containing substances in liver has an important role in the development of chronic hepatic injury. In fact we observed that induction of oxidative stress-mediated liver injury by a hepatotoxicant, such as ethanol, bacterial lipopolysaccharide (LPS), α-naphthylisothiocyanate (ANIT), chloroform, and high-fat diet, was all accompanied with profound changes in the hepatic transsulfuration reactions (Kim and Kim, 2002, 2005; Kim *et al*., 2005, 2008; Kwon *et al*., 2008). The recovery from liver injury also corresponded with retrieval of hepatic levels of sulfur-containing substances.

The present study was conducted to characterize the early changes in hepatic metabolism of sulfur-containing substances in rats treated with an acute dose of $CCl_4$, a model hepatotoxicant frequently used to develop liver injury in animal experiments. A toxic dose of $CCl_4$ increased the activity of MAT rapidly. This is contrary to the report showing a reduction of MAT activity in rats treated with $CCl_4$ repeatedly (Corrales *et al*., 1992). It has been suggested that MAT activity is regulated by cellular redox state in liver. Hepatic MAT activity was inhibited by addition of GSSG (Pajares *et al*., 1992) and also by hydroxyl radical (Sánchez-Góngora *et al*., 1997). Inactivation of MAT was reversed by GSH and other thiol-reducing agents (Corrales *et al*., 1999). In this study, hepatic MAT activity was increased significantly from 4 h after $CCl_4$ treatment, which might be associated with the elevation of GSH levels in liver. However, the induction of MAT activity did not result in an increase in SAM levels. In addition to its role as an intermediate in the transsulfuration reactions, SAM is the principal biological methyl donor and also a provider of aminopropyl groups utilized in polyamine synthesis. Therefore, SAM is not only an intermediate metabolite in methionine catabolism, but also acts as an intracellular control switch that regulates essential hepatic functions such as regeneration, differentiation, and the sensitivity of this organ to injury (Mato and Lu, 2007). The present results suggest that SAM, although its synthesis might be enhanced via induction of MAT activity and methionine availability, reduced in liver due to rapid consumption in a metabolic reaction(s) associated with induction of acute liver injury and/or regeneration process resulting from $CCl_4$ intoxication.

Hepatic GSH levels were elevated significantly from 4 h after $CCl_4$ treatment. Synthesis of GSH in liver is limited mostly by two factors; the availability of cysteine and the activity of GCL that catalyzes a rate limiting step in GSH synthesis (Lu, 1998). Hepatic cysteine was increased from 4 h following $CCl_4$ treatment, and remained at a level significantly higher than normal in this study. The GCL activity was also increased after $CCl_4$ treatment. Thus, the elevation of hepatic GSH levels was attributed to the enhanced generation of this tripeptide in hepatocytes.

In liver the cysteine concentration is tightly regulated by a balance between the rates of its synthesis, hepatic uptake from blood, and metabolism to GSH, inorganic sulfate and taurine (Stipanuk *et al*., 1992). The dose of $CCl_4$ administered to rats did not increase either CβS or CγL activity in this study. Therefore, the elevated cysteine levels may not be accounted for by the enhancement of its synthesis in liver. The hepatic levels of GSH and hypotaurine, the major metabolic products from cysteine, were increased by $CCl_4$ treatment, excluding a possibility that inhibition of cysteine catabolism to either GSH or taurine could be responsible for the increment of this sulfur-amino acid. Recently we observed that a toxic dose of $CCl_4$ elevated the hepatic cysteine contents in mice as well (Lee and Kim, 2007). The mechanism of cysteine elevation in liver remains unclear, but it seems that an increase in its uptake from plasma plays a role here. This view appears to be supported by the transient, but significant decrease in cysteine levels in plasma at 4 h following $CCl_4$ treatment.

Cysteine is catabolized oxidatively to cysteinesulfinate, which is introduced into several pathways including taurine synthesis, sulfate production, and generation of pyruvate. In the present study hepatic cysteine levels and CDO activity were elevated from 4 h after $CCl_4$ treatment, which was accompanied with an increase in hypotaurine. The induction of CDO activity is in agreement with the suggestion that the cysteine availability is a major determinant for partitioning of cysteine sulfur to either GSH, taurine or inorganic sulfates in liver (Stipanuk *et al*., 1992). Low cysteine availability would favor its utilization for the synthesis of GSH; high cysteine availability enhances its catabolism to inorganic sulfate and taurine. But in this study hepatic taurine was rather decreased significantly whereas taurine in plasma was increased. It appears that the enhancement of hepatic efflux of taurine is greater than the increase in catabolism of cysteine to taurine in acute liver injury, resulting in reduction of hepatic levels of this β-amino acid.

The increase in plasma levels of GSH and taurine is of special interest. Plasma GSH reached a level greater than ninefold of control in 24 h after $CCl_4$ treatment. Liver has a central role in the maintenance of interorgan GSH homeostasis with sinusoidal GSH efflux as the major determinant of GSH, cystine, cysteine, and thiol-disulfide status of plasma (Ookhtens and Kaplowitz, 1998). The molecular mechanisms of GSH efflux have been suggested to involve several transporters including oatpl (the sinusoidal organic anion transporter polypeptide), gsht (a putative GSH transporter on the sinusoidal side), and multidrug resistance proteins (MRPs). The enhancement of GSH efflux after $CCl_4$ treatment has been demonstrated by several authors (Irita *et al.*, 1994; Kadiiska *et al.*, 2000). Interestingly, the total antioxidant capacity of serum was also increased, but the elevation of GSH could account for only a small portion of the enhanced total antioxidant capacity in serum (Kadiiska *et al.*, 2000). In this study the elevation of plasma GSH does not appear to be associated with inhibition of its catabolism. The metabolism of GSH is mediated by $\gamma$-glutamyltransferase (GGT) that allows component amino acids to be available for intracellular GSH resynthesis and a continuous 'GSH cycling' (Whitfield, 2001). The GGT activity in kidney was not altered by $CCl_4$ treatment, indicating that the accumulation of plasma GSH might not be attributed to the destruction of this enzyme system. The dose of $CCl_4$ used was also nephrotoxic as shown by the elevation of BUN and sCr. The increase in BUN and sCr indicated that glomerular filtration rate was decreased in rats treated with $CCl_4$. However, the changes in the plasma concentration of other sulfur-containing metabolites were much smaller, suggesting that the reduction in glomerular filtration rate might not account for the marked elevation of plasma GSH. Further studies need to be conducted to identify the mechanism of GSH elevation in plasma in association with induction of acute liver injury by $CCl_4$.

In summary, the present results indicate that induction of acute liver injury by a toxic dose of $CCl_4$ is accompanied with extensive alterations in the metabolomics of sulfur-containing amino acids and related substances. The increase in hepatic MAT without a change in SAM levels suggests that the utilization of SAM is increased in a reaction other than the transsulfuration reactions. The cellular availability of cysteine and activities of both CDO and GCL are elevated, resulting in an increase in GSH and hypotaurine syntheses in liver. The major metabolites and products of the transsulfuration pathway, including methionine, cysteine, hypotaurine or taurine, and GSH, are all increased in liver and plasma after the treatment. The physiological significance of the change in the metabolomics of sulfur-containing substances and its role in the induction of liver injury warrant further studies.

## ACKNOWLEDGEMENTS

This work was supported in part by a Korea Science and Engineering Foundation (KOSEF) grant (R01-2008-000-10622-0) funded by the Ministry of Education, Science and Technology, Korea.

## List of Abbreviations

SAM: S-adenosylmethionine
MAT: methionine adenosyltransferase
SAH: S-adenosylhomocys-teine
CßS: cystathionine ß-synthase
CγL: cystathionine γ-lyase
GSH: glutathione
CDO: cysteine dioxygenase
GCL: glutamate cysteine ligase
AST: aspartate aminotransferase
ALT: alanine aminotransferase
sCr: serum creatinine
BUN: blood urea nitrogen
MDA: malondialdehyde
GGT: γ-glutamyl-transferase
ER: endoplasmic reticulum
LPS: lipopolysaccharide
ANIT: a-naphthylisothiocyanate
GSSG: glutathione disulfide
MRPs: multidrug resistance proteins
